# Single-fraction stereotactic radiosurgery versus microsurgical resection for the treatment of vestibular schwannoma: a systematic review and meta-analysis

**DOI:** 10.1186/s13643-022-02118-9

**Published:** 2022-12-12

**Authors:** Thomas Jakubeit, Sibylle Sturtz, Dorothea Sow, Wolfram Groß, Christoph Mosch, Mattea Patt, Vera Weingärtner, Jan Boström, Roland Goldbrunner, Martina Markes

**Affiliations:** 1 Institute for Quality and Efficiency in Health Care (IQWiG), Cologne, Germany; 2 Department of Radiosurgery and Stereotactic Radiotherapy, MediClin Robert Janker Clinic and MediClin MVZ Bonn, Bonn, Germany; 3 Gamma Knife Zentrum Bochum, Department of Radiotherapy and Radio-Oncology, Universitätsklinikum Marien Hospital Herne, Bochum, Germany; 4 Center for Neurosurgery, Department of General Neurosurgery, University of Cologne, Cologne, Germany

**Keywords:** Radiosurgery, Vestibular schwannoma, Benefit assessment, Systematic review

## Abstract

**Background:**

Vestibular schwannomas are benign tumours for which various treatments are available. We performed a systematic review of prospective controlled trials comparing the patient-relevant benefits and harms of single-fraction stereotactic radiosurgery (sfSRS) with microsurgical resection (MR) in patients with vestibular schwannoma.

**Methods:**

We searched for randomized controlled trials (RCTs) and non-randomized prospective controlled trials in MEDLINE, Embase, the Cochrane Central Register of Controlled Trials, and study registries (last search: 09/2021) and also screened reference lists of relevant systematic reviews. Manufacturers were asked to provide unpublished data. Eligible studies investigated at least one patient-relevant outcome. We assessed the risk of bias (high or low) at the study and outcome level. If feasible, meta-analyses were performed. We graded the results into different categories (hint, indication, or proof of greater benefit or harm).

**Results:**

We identified three non-randomized prospective controlled trials of generally low quality with evaluable data on 339 patients with unilateral vestibular schwannoma. There was an indication of greater benefit of sfSRS compared with MR for facial palsy (*OR* 0.06, 95% *CI* 0.02–0.21, *p* < 0.001, 2 studies), hearing function (no pooled estimate available, 2 studies), and length of hospital stay (no pooled estimate available, 2 studies). We found no clinically relevant differences for mortality, vertigo, headaches, tinnitus, balance function, work disability, adverse events, and health-related quality of life.

**Conclusions:**

Our systematic review indicates that sfSRS has greater benefits than MR in patients with unilateral vestibular schwannoma. However, it is unclear whether this conclusion still holds after 2 years, as long-term studies are lacking. It is also unclear whether the effects of sfSRS are similar in patients with bilateral vestibular schwannomas. Long-term prospective studies including patients with this condition would therefore be useful.

**Systematic review registration:**

The full (German language) protocol and report (Commission No. N20-03) are available on the institute’s website: www.iqwig.de/en/projects/n20-03.html

**Supplementary Information:**

The online version contains supplementary material available at 10.1186/s13643-022-02118-9.

## Background

Vestibular schwannomas (formerly called acoustic neuromas) are benign, usually slow-growing tumours that typically arise from the vestibular nerve [1]. About 8% of all intracranial tumours are vestibular schwannomas, which are unilateral in more than 90% of cases [1, 2]. The incidence is 1 to 2 cases per 100,000 population per year, and they are most common in adults aged 50 years and older [1, 2]. Symptoms include hearing loss, tinnitus, vertigo, and facial palsy [1, 2]. The exact cause is unknown. Potential risk factors include the use of cell phones, noise exposure, and low-dose radiation for benign conditions of the head and neck in childhood [2]. Bilateral vestibular schwannomas are common in patients with neurofibromatosis type 2 [3]. Different classification systems by Sterkers, House, Koos, and Samii are used for staging vestibular schwannoma based on tumour size and effects on adjacent structures [1]. The diagnosis is usually confirmed with magnetic resonance imaging (MRI) [1].

The tumour characteristics, medical history, and patient preferences are the main factors to consider when selecting a management option [1, 4–7], which include “watch and wait” as well as microsurgical resection (MR), radiation therapy, and combinations thereof [1, 2, 6]. “Watch and wait” requires regular MRI about every 12 months and is particularly an option for small, nongrowing and asymptomatic tumours [1, 2]. In contrast, surgical intervention is usually indicated for large and symptomatic tumours. Depending on the size of the tumour and the patient’s hearing ability, the retrosigmoid, translabyrinthine, or middle fossa approach may be used. Mortality, adverse events, and recurrence rates, seem to be similar between the three approaches. However, the retrosigmoid and translabyrinthine approaches appear to result in less facial nerve dysfunction, whereas the middle fossa approach appears to have better hearing preservation for small tumours [1, 2]. Radiation therapy includes stereotactic radiotherapy or single-fraction stereotactic radiosurgery (sfSRS). In stereotactic radiotherapy, focused doses of radiation are delivered over a series of treatment sessions. In contrast, sfSRS delivers a high single dose of radiation in one treatment session using multiple, nonparallel radiation beams that converge on the target lesion. The full therapeutic dose is restricted to the area where all the radiation beams overlap, whereas nontarget areas receive a much lower dose from one or a low number of the radiation beams. Therefore, accurate localization of the lesion and positioning of the patient are required during sfSRS using cobalt-60-based sources (e.g. Gamma Knife) or linear accelerator techniques (e.g. CyberKnife) [2, 8]. Usually, hospitalization is not required, and hearing function can be preserved with this method. Compared with MR, however, reinterventions may occur more frequently after sfSRS due to tumour growth [1, 2, 8].

The aim of this systematic review was to assess the patient-relevant benefits and harms of sfSRS versus MR in patients with vestibular schwannoma.

## Methods

### Protocol and methodological approach

Our review formed part of a German-language health technology assessment (HTA) of the benefits and harms of sfSRS in patients with vestibular schwannoma that was published by the Institute for Quality and Efficiency in Health Care (Institut für Qualität und Wirtschaftlichkeit im Gesundheitswesen, IQWiG) in 2021. The full (German language) protocol and report (Commission No. N20-03) are available on the institute’s website [9]. The preliminary report underwent a public commenting procedure in writing. IQWiG’s responsibilities and methodological approach are described in its methods paper [10]. Only completed studies were used, so ethical approval and patient consent were not required. We adhered to the PRISMA statement [11] throughout this manuscript.

### Eligibility criteria

Eligible studies were published and previously unpublished randomized controlled trials (RCTs) as well as non-randomized prospective controlled trials in patients with vestibular schwannoma comparing sfSRS with any kind of MR and investigating at least one predefined patient-relevant outcome. In this context, the term “patient relevant” refers to “how a patient feels, functions or survives” [12]. The detailed eligibility criteria are presented in Table 1.

**Table 1:** Eligibility criteria for studies included

Population	• Patients with vestibular schwannoma
Study intervention	• Single-fraction stereotactic radiosurgery using cobalt-60 sources (e.g. Gamma Knife) or linear accelerator techniques (e.g. CyberKnife)
Control intervention	• Any kind of microsurgical resection (e.g. retrosigmoid, translabyrinthine, or the middle fossa approach)
Patient-relevant outcomes	• Mortality • Facial palsy • Hearing function • Vertigo • Headaches • Tinnitus • Balance function • Work disability • Serious adverse events • Adverse events • Length of hospital stay • Health-related quality of life
Study design	• Randomized controlled trials • Non-randomized prospective controlled trials
Language of publication	• English or German
Publication type	• Availability of a full-text document (e.g. journal article or clinical study report)

### Information retrieval and study selection

We searched the following bibliographic databases: MEDLINE, Embase, the Cochrane Central Register of Controlled Trials, the Cochrane Database of Systematic Reviews, and the Health Technology Assessment Database. The peer-reviewed search strategy included a combination of subject headings and free text, with terms such as “vestibular schwannoma” and “stereotactic radiosurgery” (see Additional file 1 for the full search strategy). In addition, we searched ClinicalTrials.gov and the International Clinical Trials Registry Platform Search Portal. The last search was conducted on 23 September 2021. The reference lists of relevant systematic reviews were scrutinized to identify further studies. We also asked medical device manufacturers to provide unpublished studies in order to obtain the most complete data set possible (see Additional file 1 for the full list of manufacturers). In addition, persons and parties who had submitted comments on the preliminary version of the IQWiG report were asked to provide any additional relevant studies.

Two reviewers independently screened titles and abstracts of the citations retrieved to identify potentially eligible primary and secondary publications. The full texts of these articles were obtained and independently evaluated by the same reviewers. All documents retrieved from non-bibliographical sources were also screened for eligibility or relevant information on studies. Disagreements were resolved by consensus.

### Data extraction

The data extraction and risk-of-bias assessment procedures were always conducted by one person and checked by another; disagreements were resolved by consensus. Details of the studies were extracted using standardized tables. We contacted the study authors if relevant data were missing (including statistical analyses).

We extracted information on the following:

1. Study characteristics, including study design, number of centres, number of patients included, length of follow-up, location, and period in which the study had been conducted
2. Characteristics of the study participants, including inclusion and exclusion criteria, age, sex, tumour characteristics, hearing function, and facial function at baseline
3. Outcomes and type of outcome measures: outcomes as presented in Table 1
4. Risk-of-bias items (see below)

### Risk-of-bias assessment

Using the IQWiG methods [10], we assessed the risk of bias (high or low) at the study and outcome level. For RCTs, we planned the assessment of the following items at the study level: generation of a randomization sequence, allocation concealment, blinding of patients and treating staff, reporting of all relevant outcomes independently of results, and other aspects. A high risk of bias at the study level generally led to a high risk of bias at the outcome level. Otherwise, the following outcome-specific items were assessed: blinding of outcome assessors, appropriate application of the intention-to-treat principle, reporting of individual outcomes independently of results, and other aspects.

For non-randomized prospective controlled trials, we assessed the following items at the study level: temporal parallelism of the intervention groups, comparability of intervention groups and appropriate consideration of relevant prognostic factors, blinding of patients and treating staff, reporting of all relevant outcomes independently of results, and the absence of other factors potentially causing bias. Due to the study design (especially lack of randomization) of non-randomized prospective controlled studies, the risk of bias at the study level is generally considered high. Thus, outcome-specific items were not assessed, since a high risk of bias at the study level generally led to a high risk of bias at the outcome level.

In a further step, we assessed the qualitative certainty of study results and, depending on the results of the risk-of-bias assessment, graded this qualitative certainty as moderate or high for RCTs and as low or very low for non-randomized prospective controlled trials.

Using the IQWiG methods [10], we planned to grade the results of the (meta-)analysis into different categories: “proof”, “indication”, or “hint” (or none of these categories) of greater benefit of the test intervention. In short, proof of greater benefit of the test intervention is inferred if a meta-analysis of at least 2 studies with a high qualitative certainty of results shows a statistically significant effect favouring the test intervention. An indication of greater benefit is inferred if a single study with a high qualitative certainty of results shows a statistically significant effect favouring the test intervention, or a meta-analysis of studies with a moderate qualitative certainty of results shows a statistically significant effect favouring the test intervention. A hint of greater benefit is inferred if either a single study with a moderate qualitative certainty of results or a meta-analysis of studies with a low qualitative certainty of results shows a statistically significant effect favouring the intervention. No proof (nor indication nor hint) of greater benefit or harm is inferred if there are no statistically significant differences between the test and control interventions, if relevant heterogeneity exists, or if no suitable data are available.

Based on a single study with very low qualitative certainty of results, a hint of greater benefit is also inferred in the case of a very large magnitude of an effect that cannot be explained by bias alone (“dramatic effect”).

In addition to the IQWiG methods, we also applied the GRADE approach (Grading of Recommendations Assessment, Development and Evaluation) in order to describe the certainty of the evidence [13].

### Data analysis

Using the IQWiG methods [10], we planned to analyse different study designs together, taking into account the qualitative certainty of the results when grading the results. Odds ratios (ORs) were calculated to compare dichotomously measured outcomes, and mean differences (MDs) were calculated to compare continuously measured outcomes. For all effect estimates, 95% confidence intervals (CIs) were reported. If feasible and meaningful, data were pooled by means of meta-analyses. If only 2 studies were available, a fixed-effect model with inverse variance (or according to Mantel-Haenszel [14]) was used to combine the study results. If relevant heterogeneity [15] was present (*p* < 0.05), no overall effect estimate was calculated. The results of the meta-analysis were presented in a forest plot. A *p*-value of < 0.05 was considered statistically significant. We also planned subgroup analyses for age and sex and for patients with neurofibromatosis type 2.

If results for different time points were available, the last one was used for the analysis (if there were no inconsistencies compared with earlier time points), as treatment of vestibular schwannomas is aimed at reducing morbidity and improving quality of life in the long term.

## Results

### Information retrieval

**Fig. 1:**
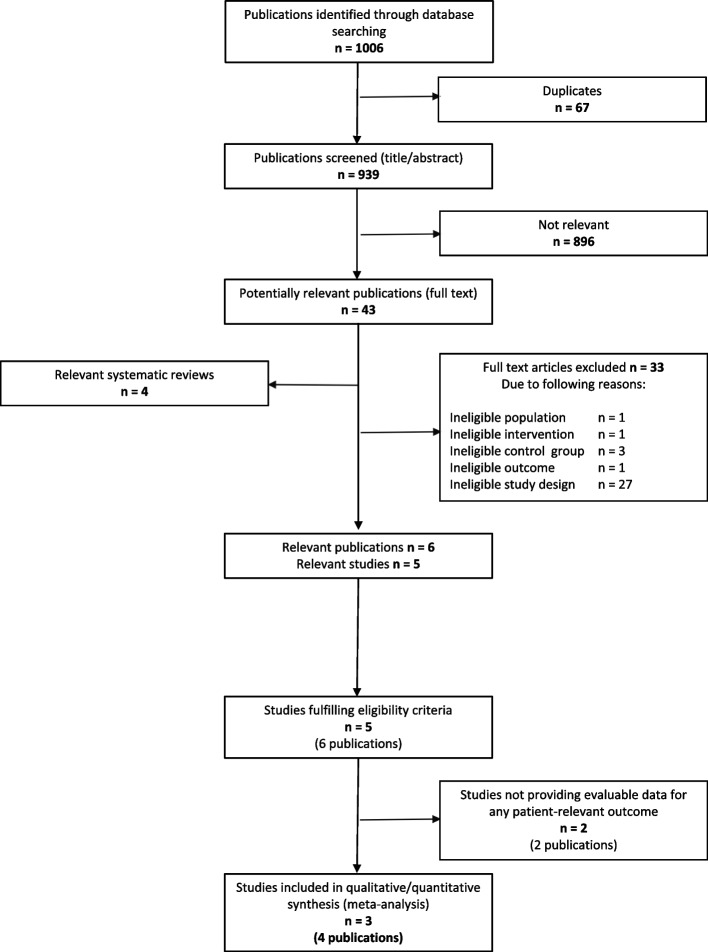
Flowchart of study selection

A total of 5 eligible studies (6 publications) were identified from 939 references (without duplicates) retrieved from bibliographic databases. Details of the study selection process from bibliographic databases are shown in Fig. 1. No additional studies were identified by reviewing the reference lists of relevant systematic reviews. A total of 6 of the 11 manufacturers sent us a response stating that there were no unpublished studies. Moreover, the written comments on the preliminary version of the IQWiG report did not provide any new studies. The list of excluded studies is shown in Additional file 2.

The 5 eligible studies (all non-randomized prospective controlled trials) fulfilled the eligibility criteria. However, Di Maio (2009) [16] and Wagner (2011) [17] were excluded from the analysis, as evaluable results were missing. In Di Maio (2009), a combined analysis of both groups was provided for patients with sfSRS and radiotherapy. Subgroup analyses for patients treated with sfSRS are not included in the publication and could not be provided by the authors. In Wagner (2011), the treatment decision was based on tumour size. No analysis stratified by tumour size or any other adjusted analysis was available. Patient characteristics allowing an assessment of the comparability of groups were not available. We ultimately included 3 studies reporting evaluable results on patient-relevant outcomes (Carlson 2021 [18, 19], Myrseth 2009 [20], and Pollock 2006 [21]).

### Characteristics of studies included

Table 2 presents the main characteristics of the 3 studies included.

**Table 2:** Characteristics of studies included

Study	Study design	Participants included	Intervention and control	Location and study period	Follow-up	Patient-relevant outcomes
Carlson 2021^a^ [18, 19]	Non-randomized prospective controlled trial, single centre	166	sfSRS MR	USA 2005–2019	2.1 years (mean)	Facial palsy Hearing function Vertigo Headaches Tinnitus Balance function Adverse events Health-related quality of life
Myrseth 2009 [20]	Non-randomized prospective controlled trial, single centre	91	sfSRS MR	Norway 2000–2007	2.0 years	Mortality Facial palsy Hearing function Vertigo Tinnitus Balance function Work disability Adverse events Length of hospital stay Health-related quality of life
Pollock 2006 [21]	Non-randomized prospective controlled trial, single centre	82	sfSRS MR	USA 2000–2002	42 months (mean)	Facial palsy Hearing function Vertigo Headaches Adverse events Length of hospital stay Health-related quality of life

*MR* microsurgical resection, *sfSRS* single-fraction stereotactic radiosurgery

^a^this is a 3-arm study. Data on the study arm with the treatment strategy of “watch and wait” are not presented

The 3-arm, single-centre US study by Carlson (2021) [18, 19] included patients with unilateral vestibular schwannoma who completed both a baseline survey before the intervention and at least one post-treatment survey. Patient preference was the determining factor for assignment to treatment groups. Overall, 244 of initially 313 patients (78%) met the inclusion criteria. Of these patients, 48 (20%) were treated with sfSRS, 118 (48%) with MR, and 78 (32%) were only watched. The mean follow-up duration was 2.1 years. Only the first 2 treatment groups are considered below. Patients in the MR group were younger on average (*p* < 0.001; IQWiG’s own calculation) and had larger tumours (*p* = 0.002; IQWiG’s own calculation) than those in the sfSRS group. The groups were comparable at baseline with regard to other patient characteristics (e.g. sex, hearing function, and facial paralysis). The sfSRS was performed with cobalt-60 gamma radiation sources. The median tumour margin dose was 12.5 gray (Gy). For the microsurgical procedure, the retrosigmoid approach was used most frequently (*n* = 71; 60%), followed by the translabyrinthine (*n* = 45; 38%), middle fossa (*n* = 1; 1%), and transotic approach (*n* = 1; 1%). Subtotal resection was performed in 18 of the 118 cases (15%). See Table 1 in Additional file 3 for details on patient characteristics.

The single-centre Norwegian study by Myrseth (2009) [20] included patients who had a unilateral, vestibular schwannoma with a diameter of ≤ 2.5 cm that was considered to require intervention because of tumour growth or a size of > 2.0 cm in the cerebellopontine angle. Patients with neurofibromatosis type 2 were excluded. Patient preference was the determining factor for assignment to treatment groups unless the surgical option was contra-indicated. In addition, some patients had a treatment prescription from referring neurosurgical centres. Originally, the study was planned as a randomized trial; due to patient refusal regarding randomization, allocation was performed as described. The study evaluated 88 of 91 patients. Of these, 60 were treated with sfSRS and 28 with MR. The follow-up period was 2 years. Patients in the sfSRS group were older on average than those in the MR group (57.5 years versus 52.5 years, *p* = 0.06). At baseline, all patients included had normal facial function (House-Brackmann scale), and 43% had functional hearing. With regard to symptoms, 83% suffered from tinnitus, 48% from vertigo, and 39% from balance dysfunction. sfSRS was performed under local anaesthesia using cobalt-60 radiation sources. The tumour margin dose was 12 Gy. MR was performed by suboccipital craniotomy with a free bone flap, which was subsequently reinserted. In one patient with severe vertigo, resection was performed using a translabyrinthine approach. In 5 cases in the MR group, the tumour could not be completely removed. See Table 2 in Additional file 3 for details on patient characteristics.

The single-centre US study by Pollock (2006) [21] included patients who had unilateral vestibular schwannoma with a diameter of < 3 cm. Patients with neurofibromatosis type 2, tumour recurrence, or who were unsuitable for resection were excluded. Overall, 82 of initially 162 patients (51%) participated in the study. Patient preference was the determining factor for assignment to treatment groups: 46 patients were treated with sfSRS and 36 with MR. Follow-up duration averaged 42 months across both treatment arms, with a minimum of 12 months and a maximum of 62 months. Patients in the MR group were on average younger than those in the sfSRS group (48.2 years versus 53.9 years, *p* = 0.03). Both groups were comparable at baseline with regard to other patient characteristics (e.g. sex, hearing function, tinnitus, vertigo, and tumour size). sfSRS was performed with cobalt-60 gamma radiation sources. The tumour margin dose was 12.2 Gy. The microsurgical procedure was selected based on patient preference, hearing function, and tumour size. The retrosigmoid approach was used most frequently (*n* = 25; 69%), followed by the translabyrinthine (*n* = 9; 25%) and middle fossa approach (*n* = 2; 6%). In 3 of the 36 cases, the tumour could not be completely removed. See Table 3 in Additional file 3 for details on patient characteristics.

### Risk of bias and qualitative certainty of results

Table 3 presents the risk of bias of the studies included. Due to the study design (especially lack of randomization) of the included non-randomized prospective controlled studies, the risk of bias at the study level was considered high. Thus, outcome-specific items were not assessed, since a high risk of bias at the study level generally led to a high risk of bias at the outcome level. For all studies, we graded the qualitative certainty of results as very low, since either comparability of intervention groups and appropriate consideration of relevant prognostic factors were lacking (Myrseth 2009 and Pollock 2006), or it remained unclear for what proportion of patients the survey was at least partially retrospective (Carlson 2021). Based on the very low qualitative certainty of results, a hint of greater benefit was only inferred in the case of a very large magnitude of an effect that could not be explained by bias alone (dramatic effect).

**Table 3:** Risk of bias of studies included

Study	Parallelism of groups	Comparability of groups	Blinding		Selective reporting improbable	Absence of other factors potentially causing bias	Risk of bias: study level	Risk of bias: outcome level
			Patients	Treating staff				
Carlson 2021 [18, 19]	Yes	Yes	No	No	Unclear	No^a^	High	High
Myrseth 2009 [20]	Yes	No^b^	No	No	Unclear	Yes	High	High
Pollock 2006 [21]	Yes	No^c^	No	No	Unclear	Yes	High	High

^a^patients were screened for inclusion criteria between 2005 and 2019, although the study start date was reported as 2014. Thus, it remains unclear for what proportion of patients the survey was at least partially retrospective (“obtained by medical record review”). Based on all publications, it cannot be assumed that this is a retrospective study

^b^although the essential data are available at baseline (age, sex, symptom severity, and tumour size), the groups differ in age by an average of 5.0 years (*p* = 0.06)

^c^although the essential data are available at baseline (age, sex, symptom severity, and tumour size), the groups differ statistically significantly in age by an average of 5.7 years (*p* = 0.03)

### Effects of sfSRS versus MR

Data on mortality were available in one study (Myrseth 2009). No deaths occurred during the study period of 2 years. There was thus no indication of greater or lesser benefit of sfSRS compared with MR for mortality.

**Fig. 2:**
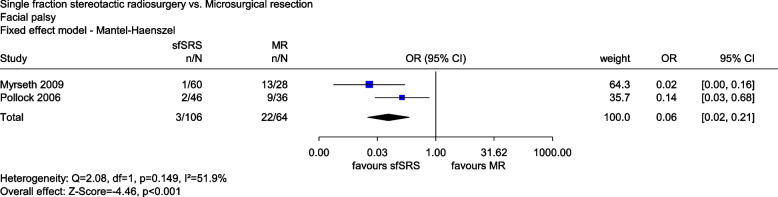
Forest plot of facial palsy, sfSRS vs. MR. Abbreviations: CI confidence interval, n number of events, N number of patients, MR microsurgical resection, OR odds ratio, sfSRS single-fraction stereotactic radiosurgery, vs versus

Myrseth (2009) and Pollock (2006) provided data on facial palsy measured with the House-Brackmann score. Since only grade 1 corresponds to normal facial function, the remaining grades 2 to 6 were operationalized as facial palsy. The meta-analysis of the last time points (24 months and 42 months [mean]) showed a statistically significant effect in favour of sfSRS compared with MR (*OR*: 0.06, 95% *CI*: [0.02, 0.21], *p* < 0.001, see Fig. 2). The odds of suffering facial palsy were about 17 times lower when treated with sfSRS compared with treatment with MR. Such a very large magnitude of an effect cannot be explained by bias alone (dramatic effect). There was thus an indication of greater benefit of sfSRS compared with MR for facial palsy.

The results for hearing function were reported in 3 studies. Hearing was considered functionally preserved if grade A or B was maintained according to the Gardner-Robertson scale or according to classification of the American Academy of Otolaryngology — Head and Neck Surgery (AAO-HNS). No overall effect estimate could be calculated due to insufficient presentation of results and different operationalizations of this outcome. The results of Myrseth (2009) (Gardner-Robertson scale) showed a statistically significant difference in favour of sfSRS compared with MR at 24 months (*OR*: 22.93, 95% *CI*: [1.33, 396.64], *p* = 0.002, see Table 1 in Additional file 4). The odds of preserving functional hearing were about 23 times higher with treatment with sfSRS compared with MR. The results of Pollock (2006) (AAO-HNS classification) showed a statistically significant difference at 42 months (mean) in favour of sfSRS compared with MR (*p* < 0.001, no further information available, see Table 1 in Additional file 4). In Carlson (2021), hearing was assessed using a Likert scale with a range of 1 (normal hearing) to 10 (completely deaf). There was a statistically significant difference at 2.1 years (mean) in favour of sfSRS compared with MR (mean difference [MD]: −1.60, 95% *CI*: [−2.63, −0.57], *p* = 0.002, see Table 2 in Additional file 4). However, preservation of functional hearing cannot be assessed with this instrument, so the results of the Likert scale should be considered as a supplement to the relevant analyses in Myrseth (2009) and Pollock (2006). In summary, Myrseth (2009) in particular showed a very large magnitude of an effect that could not be explained by bias alone (dramatic effect). There was thus an indication of greater benefit of sfSRS compared with MR for hearing function.

**Fig. 3:**
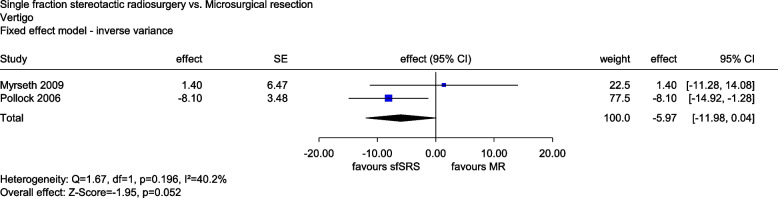
Forest plot of vertigo, sfSRS vs. MR. Abbreviations: CI confidence interval, MR microsurgical resection, SE standard error, sfSRS single-fraction stereotactic radiosurgery, vs versus

Two studies provided data on vertigo. Data were collected in Myrseth (2009) using a visual analogue scale and in Pollock (2006) using the Dizziness Handicap Inventory (range of values in each case from 0 to 100, higher values corresponding to greater perceived symptoms). Both instruments were found to be sufficiently similar to be combined meta-analytically. This showed a numerical advantage of sfSRS compared with MR at the last follow-up visits (24 months and 42 months [mean]), but no statistically significant difference between groups (*MD*: −5.97, 95% CI: [−11.98, 0.04]; *p* = 0.052, see Fig. 3). There was thus no indication of greater or lesser benefit of sfSRS compared with MR for vertigo.

Data on headaches were available from 2 studies. Because of different operationalizations of this outcome, no overall effect estimate could be calculated. In Carlson (2021), the outcome was assessed using a Likert scale with a range of 1 to 10 (higher scores correspond to greater perceived symptoms). Pollock (2006) used the headache survey, which is composed of questions about the frequency, duration, intensity, treatment, and disability associated with headaches. According to the rating, the range of values is between 1 and 20 (higher values correspond to greater perceived symptoms). Neither Carlson (2021) at 2.1 years (mean) nor Pollock (2006) at 42 months (mean) showed a statistically significant difference between groups (*p* = 0.871 and *p* = 0.29, see Table 3 in Additional file 4). There was thus no indication of greater or lesser benefit of sfSRS compared with MR for headaches.

**Fig. 4:**
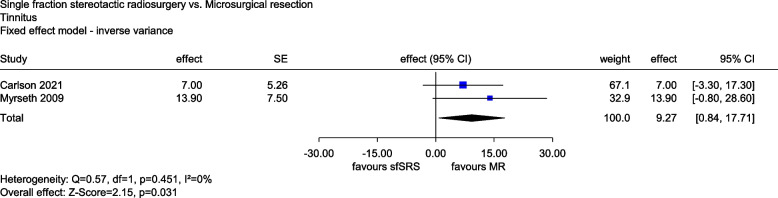
Forest plot of tinnitus, sfSRS vs. MR. Abbreviations: CI confidence interval, MR microsurgical resection, SE standard error, sfSRS single-fraction stereotactic radiosurgery, vs versus

Data on tinnitus were available from 2 studies. In Carlson (2021), this outcome was assessed using a Likert scale with a value range of 1 to 10 (higher values correspond to greater perceived symptoms). In Myrseth (2009), the survey was conducted using a visual analogue scale with a range of values from 0 to 100 (higher values correspond to greater perceived symptoms). Since, with the exception of the value range, both scales are comparable, the value range in Carlson (2021) was multiplied by 10. Meta-analysis of the last time point (2.1 years [mean] or 24 months) showed a statistically significant difference to the disadvantage of sfSRS compared with MR (*MD*: 9.27, 95% *CI*: [0.84, 17.71], *p* = 0.031, see Fig. 4). However, a mean difference of about 9 on a scale of 1 to 100 is not of a magnitude that cannot be explained by bias alone (no dramatic effect). There was thus no indication of greater or lesser benefit of sfSRS compared with MR for tinnitus.

Data on balance function were available from Myrseth (2009). After 24 months, 45.0% of patients in the sfSRS group and 50.0% in the MR group reported that they suffered from balance disorders. The difference was not statistically significant (no further information available, see Table 4 in Additional file 4). There was thus no indication of greater or lesser benefit of sfSRS compared with MR for balance function.

Data on work disability were available from 1 study. In Myrseth (2009) (at baseline and 24 months), patients were asked whether they were working, on sick leave, disability, or retired. This study lacks the specific operationalizations of the patient characteristics that would allow them to be distinguished from one another. Regardless of this, there were no statistically significant differences between the groups with respect to the 4 options at 24 months (*p* = 0.924, see Table 5 in Additional file 4). There was thus no indication of greater or lesser benefit of sfSRS compared with MR for this outcome.

No data on serious adverse events (SAEs) were available from the 3 studies. There was thus no indication of greater or lesser benefit of sfSRS compared with MR for SAEs.

Data on adverse events (AEs), including treatment complications and reinterventions, were available from 2 studies. In Myrseth (2009), AEs were not reported for any of the 60 patients treated with sfSRS. After MR, 9 AEs occurred in 28 patients. These included various forms of plastic surgery to correct postoperative facial palsy (*n* = 5), cerebrospinal fluid leakage requiring reoperation (*n* = 2), asymptomatic small haematoma in the resection cavity found on the computed tomographic scan (*n* = 1), and hoarseness resolving in a few weeks (*n* = 1). In Pollock (2006), 3 AEs (6.5%) occurred after sfSRS in 46 patients. These were increasing ataxia (*n* = 2) and trigeminal neuralgia (*n* = 1). After MR, 13 AEs occurred in 36 patients. These included cerebrospinal fluid leakage (*n* = 5), tarsorrhaphy (*n* = 5), gold weight placement for eye protection (*n* = 1), deep vein thrombosis (*n* = 1), and wound infection (*n* = 1). The overall rate of AEs was considered to be uninterpretable, and therefore, an overall effect estimate was not calculated. On the one hand, the severity of individual AEs cannot be assessed on the basis of the available data; on the other, it cannot be ruled out that multiple events occurred in one person. Furthermore, it also remains unclear whether a systematic approach was used to monitor AEs. Consequently, no conclusion on benefits or harms can be drawn for AEs.

**Fig. 5:**
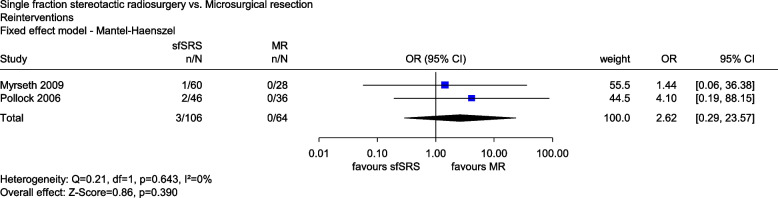
Forest plot of reinterventions, sfSRS vs. MR. Abbreviations: CI confidence interval, n number of events, N number of patients, MR microsurgical resection, OR odds ratio, sfSRS single-fraction stereotactic radiosurgery, vs versus

In both studies, reinterventions were performed only after sfSRS. In Myrseth (2009), 1 of 60 patients (1.7%) underwent MR due to tumour growth within 24 months; in Pollock (2006), 2 of 46 patients (4%) were affected within 42 months (mean). Meta-analysis showed no statistical difference between the 2 groups (*OR*: 2.62, 95% *CI*: [0.29, 23.57], *p* = 0.390, see Fig. 5). There was thus no indication of greater or lesser benefit of sfSRS compared with MR for AEs, including treatment complications and reinterventions.

Data on length of hospital stay were available from 2 studies. Because of the insufficient presentation of results, no overall effect estimate could be calculated. In Myrseth (2009), there was a statistically significant effect in favour of sfSRS (mean [min, max]: 2.5 [2, 5] days compared to 12.5 [10, 30] days, *p* < 0.001). In Pollock (2006), sfSRS was performed as an outpatient procedure. The average length of hospital stay after MR was 5.1 days (no further information available). In summary, the results of both studies showed very large effects that cannot be explained by bias alone (dramatic effect). There was thus an indication of greater benefit of sfSRS compared with MR for length of hospital stay.

Data on health-related quality of life (HrQoL) were available from 3 studies. In Carlson (2021), the authors measured this outcome using the Penn Acoustic Neuroma Quality-of-Life (PANQOL) scale. The total score is based on 7 domain scores; each cover a range of values from 0 to 100, and higher values correspond to lower perceived symptom. After 2.1 years (mean), the total score showed a numerical advantage of sfSRS compared with MR, but no statistically significant difference between groups (*MD*: 5.00; 95% *CI*: [−3.41, 13.41], *p* = 0.242, see Table 6 in Additional file 4). There was thus no indication of greater or lesser benefit of sfSRS compared with MR for the PANQOL scale.

In Myrseth (2009), HRQoL was measured using the Glasgow Benefit Inventory (GBI). The total score and the 3 domain scores each cover a range of values from −100 to 100, and higher values correspond to lower perceived symptom. After 2 years, the total score showed a statistical significant effect in favour of sfSRS compared with MR (*MD*: 13.90, 95% *CI*: [3.02, 24.78], *p* = 0.013, see Table 7 in Additional file 4). However, a mean difference of about 14 on a scale of −100 to 100 is not of a magnitude that cannot be explained by bias alone (no dramatic effect). There was thus no indication of greater or lesser benefit of sfSRS compared with MR for the GBI.

In Pollock (2006), HRQoL was measured using the tinnitus survey and the 36-item short-form health survey (SF-36). The tinnitus survey assesses the extent to which tinnitus affects HRQoL. The instrument covers a range of values from 0 to 100, and higher values correspond to a greater perceived symptom. After 42 months (mean), there was no statistically significant difference between groups (*p* = 0.29, see Table 8 in Additional file 4). The SF-36 has 2 summary scores. The mental component summary (MCS) and the physical component summary (PCS) each have a range of values from 0 to 100, and higher values correspond to a lower perceived symptom. After 42 months (mean), the MCS showed a numerical advantage of sfSRS (*MD*: 3.30, 95% *CI*: [−0.41, 7.01], *p* = 0.080), and the PCS showed a numerical disadvantage of sfSRS (*MD*: –0.70, 95% *CI*: [−5.35, 3.95], *p* = 0.765). However, there was no statistically significant difference between groups (see Table 9 in Additional file 4). There was thus no indication of greater or lesser benefit of sfSRS compared with MR for the tinnitus survey and the SF-36.

In summary, there was no indication of greater or lesser benefit of sfSRS compared with MR for HRQoL.

No study reported results on subgroup characteristics. We therefore did not perform subgroup analyses. A summary of results is presented in Table 4. An overview of the key findings according to the GRADE methods is provided in Additional file 5.

**Table 4:** Summary of results

Outcome	Results	Grading of results
Mortality	Information on mortality was available descriptively in one study involving 91 patients. No deaths occurred during the study period of 2 years	(⇔)
Facial palsy	*OR*: 0.06, 95% *CI*: [0.02, 0.21], *p* < 0.001, 2 studies	⇗^a^
Hearing function^b^	Three studies showed statistically significant difference in favour of sfSRS compared with MR	⇗^a^
Vertigo	*MD*: −5.97, 95% *CI*: [−11.98, 0.04], *p* = 0.052, 2 studies	⇔
Headaches^b^	Two studies found no statistically significant difference between groups	⇔
Tinnitus	*MD*: 9.27, 95% *CI*: [0.84, 17.71], *p* = 0.031, 2 studies	⇔^c^
Balance function	One study found no statistically significant difference between groups	⇔
Work disability	One study found no statistically significant difference between groups	⇔
Serious adverse events	No study reported this outcome	–
Adverse events (reinterventions)	*OR*: 2.62, 95% *CI*: [0.29, 23.57], *p* = 0.390, 2 studies	(⇔)
Length of hospital stay^b^	Two studies showed statistically significant difference in favour of sfSRS compared with MR	⇗^a^
Health-related quality of life^b^	Two studies found no statistically significant difference between groups. However, one study showed a statistical significant effect in favour of sfSRS compared with MR (*MD*: 13.90, 95% *CI*: [3.02, 24.78], *p* = 0.013)	⇔^d^

*CI* confidence interval, *MD* mean difference, *MR* microsurgical resection, *OR* odds ratio, *sfSRS* single-fraction stereotactic radiosurgery

^a^the studies showed a very large magnitude of an effect that could not be explained by bias alone

^b^it was not possible to pool the data due to the way they were reported

^c^a mean difference of about 9 on a scale of 1 to 100 is not of a magnitude that cannot be explained by bias alone (no dramatic effect). There was thus no indication of greater or lesser benefit of sfSRS compared with MR for this outcome

^d^a mean difference of about 14 on a scale of −100 to 100 is not of a magnitude that cannot be explained by bias alone (no dramatic effect). There was thus no indication of greater or lesser benefit of sfSRS compared with MR for this outcome. ⇗, indication of greater benefit of sfSRS compared with MR.⇔, no indication of greater or lesser benefit of sfSRS compared with MR. (⇔), no indication of greater or lesser benefit of sfSRS compared with MR; the 95% CI for the relative effect is so imprecise that neither a halving nor a doubling of the effect can be excluded

## Discussion

### Summary of findings

This systematic review of sfSRS versus MR in patients with vestibular schwannoma showed some advantages of sfSRS with regard to facial palsy, hearing function, and length of hospital stay. We found no clinically relevant differences for any other outcome. These includes mortality, vertigo, headaches, tinnitus, balance function, work disability, (serious) adverse events, and health-related quality of life.

### Comparison with previous research

We identified 4 previous systematic reviews on sfSRS [22–25]. Differences in results are based particularly on inclusion criteria regarding study type [22, 23], operationalization of outcomes [22], and inclusion of only one outcome [24], as described below.

In Liu 2015 [22], a statistically significant difference in favour of sfSRS compared with MR was shown for AEs. This deviates from our findings and is probably due to the fact that first, the authors included the retrospective study Régis (2002), and second, the results on facial palsy were not considered separately but classified as an AE and evaluated together with other AEs. For functional hearing, the authors reported a statistically significant difference in favour of sfSRS compared to MR. The results from Régis (2002) were also used for this evaluation. No results were available from the Cochrane review by Muzevic (2014) [23], as no RCTs were identified. The inclusion criteria did not include other study types. The systematic review by Sabab (2018) [24] only considered one outcome (postoperative headache), without restricting the study type. There are no studies listed in the publication that meet the inclusion criteria of our review. In addition to Myrseth (2009) and Pollock (2006), 4 retrospective studies were included in Wolbers (2013) [25]. No meta-analyses are presented, only results for individual studies.

### Strengths and limitations

The systematic approach is the major strength of our review. We searched numerous bibliographic databases and study registries for published and ongoing studies. We also asked medical device manufacturers for unpublished studies, and contacted authors for additional information, including statistical analyses. Furthermore, we assessed the qualitative certainty of study results. The results are presented both in detail and as an overview of the key findings according to the GRADE methods.

Our review has some limitations that are not based on the methodological approach but on the included studies. First is the lack of long-term studies, i.e. studies lasting more than 2 years. Because treatment of vestibular schwannoma aims to reduce morbidity and improve HrQoL in the long term, data beyond the 2-year period using prospective comparative studies would be useful, particularly for hearing function, reinterventions, and HRQoL. For example, in a retrospective comparative study with a follow-up of 5 years, hearing function decreased continuously after sfSRS, whereas only a slight decrease was observed after MR [26]. Second, our review did not include a broad range of patients or treatments. For instance, it only considered patients with unilateral vestibular schwannoma, as 2 of the 3 studies we included explicitly excluded patients with bilateral vestibular schwannomas, and this patient group was not mentioned in the third study. Moreover, we only considered one comparator intervention (MR); other options, such as “watch and wait”, fractionated radiotherapy, or the combination therapy of MR and sfSRS was not considered.

### Implications for future research

The limitations of this review indicate that future studies should be longer term and also include patients with bilateral vestibular schwannomas.

In addition, non-randomized prospective controlled trials should adequately consider relevant prognostic factors in the statistical analyses. In the studies included in our review, patients were largely allocated to treatment arms according to their wishes. As a result, there were differences in group sizes and between treatment groups with respect to prognostic factors, such as age or tumour size (only the Carlson (2021) study considered prognostic factors in the statistical analyses). Such differences would not arise with randomized assignment; however, this study design seems unrealistic in this certain condition.

We did not identify any relevant study registry entries for our research question. This also applies to the studies included in our review. In this context, it is worth recalling the Declaration of Helsinki. Since 2008, every study involving human subjects must be registered in a publicly accessible database before the first subject is recruited [27].

## Conclusion

Our systematic review indicates that sfSRS has greater benefits than MR in patients with unilateral vestibular schwannoma. However, it is unclear whether this conclusion still holds after a period of 2 years, as long-term studies are lacking. It is also unclear whether the effects are similar in patients with bilateral vestibular schwannomas. Long-term prospective studies including patients with this condition would therefore be useful.

## Supplementary Information

**Additional file 1.** Search strategies applied and manufacturers contacted.

**Additional file 2.** List of excluded studies.

**Additional file 3.** Details on patient characteristics.

**Additional file 4.** Detailed results not presented in the manuscript.

**Additional file 5.** Overview of key findings according to GRADE.

## Data Availability

All data used in this article are available in the full German-language report published on the IQWiG website [9]: www.iqwig.de/en/projects/n20-03.html.

## Abbreviations

AAO-HNS: American Academy of Otolaryngology-Head and Neck Surgery
AE: Adverse event
CI: Confidence interval
GBI: Glasgow Benefit Inventory
GRADE: Grading of Recommendations Assessment, Development and Evaluation
Gy: gray
HRQoL: Health-related quality of life
HTA: Health technology assessment
IQWiG: Institute for Quality and Efficiency in Health Care (Institut für Qualität und Wirtschaftlichkeit im Gesundheitswesen)
MCS: Mental component summary
MD: Mean difference
MR: Microsurgical resection
OR: Odds ratio
PANQOL: Penn Acoustic Neuroma Quality of Life
PCS: Physical component summary
PRISMA: Preferred Reporting Items for Systematic reviews and Meta-Analyses
RCT: Randomized controlled trial
SF-36: 36-item short-form health survey
sfSRS: Single-fraction stereotactic radiosurgery
