# Supplementary material for: Single-fraction stereotactic radiosurgery versus microsurgical resection for the treatment of vestibular schwannoma: a systematic review and meta-analysis
Source: Syst Rev. 2022 Dec 12;11:265. doi: 10.1186/s13643-022-02118-9 (PMC9743510; doi:10.1186/s13643-022-02118-9)
Supplement: Supplementary file 1 — Additional file 1. Search strategies applied and manufacturers contacted. [file 13643_2022_2118_MOESM1_ESM.docx]

**Additional file 1: Search strategies applied and manufacturers contacted**

**Search strategies**

Medline via Ovid

- Ovid MEDLINE(R) 1946 to September 21, 2021

Search filters:

RCTs: Lefebvre [1] – Cochrane Highly Sensitive Search Strategy for identifying randomized trials in MEDLINE: sensitivity-maximizing version (2008 revision)

- Non‐randomized studies: Waffenschmidt [2] – Search filter with best sensitivity for controlled NRS (Ovid MEDLINE, adapted from PubMed)

| # | Searches |
| --- | --- |
| 1 | Neuroma, Acoustic/ |
| 2 | Cerebellopontine Angle/ |
| 3 | (vestibular* adj1 schwannoma*).ti,ab. |
| 4 | (acoustic adj1 (neuroma* or tumor*)).ti,ab. |
| 5 | ((cerebellopontine* adj1 angle*) and (tumor* or tumour*)).ti,ab. |
| 6 | or/1-5 |
| 7 | Radiosurgery/ |
| 8 | (gamma* adj1 knife*).ti,ab. |
| 9 | (linac* or (linear* adj1 accelerator*)).ab,ti. |
| 10 | (cyber knife* or cyberknife*).ab,ti. |
| 11 | (stereotactic* adj1 radiosurg*).ti,ab. |
| 12 | or/7-11 |
| 13 | and/6,12 |
| 14 | exp cohort studies/ or exp epidemiologic studies/ or exp clinical trial/ or exp evaluation studies as topic/ or exp statistics as topic/ |
| 15 | ((control and (group* or study)) or (time and factors) or program or survey* or ci or cohort or comparative stud* or evaluation studies or follow-up*).mp. |
| 16 | or/14-15 |
| 17 | (animals/ not humans/) or comment/ or editorial/ or exp review/ or meta analysis/ or consensus/ or exp guideline/ |
| 18 | hi.fs. or case report.mp. |
| 19 | or/17-18 |
| 20 | 16 not 19 |
| 21 | Randomized Controlled Trial.pt. |
| 22 | Controlled Clinical Trial.pt. |
| 23 | (randomized or placebo or randomly or trial or groups).ab. |
| 24 | drug therapy.fs. |
| 25 | or/21-24 |
| 26 | exp animals/ not humans/ |
| 27 | 25 not 26 |
| 28 | cochrane database of systematic reviews.jn. |
| 29 | (search or MEDLINE or systematic review).tw. |
| 30 | meta analysis.pt. |
| 31 | or/28-30 |
| 32 | (27 or 31) not (comment or editorial).pt. |
| 33 | 13 and (20 or 32) |
| 34 | 33 and (english or german).lg. |

MEDLINE(R) Epub Ahead of Print and In-Process & Other Non-Indexed Citations via Ovid to September 22, 2021

| # | Searches |
| --- | --- |
| 1 | (vestibular* adj1 schwannoma*).ti,ab. |
| 2 | (acoustic adj1 (neuroma* or tumor*)).ti,ab. |
| 3 | ((cerebellopontine* adj1 angle*) and (tumor* or tumour*)).ti,ab. |
| 4 | or/1-3 |
| 5 | (gamma* adj1 knife*).ti,ab. |
| 6 | (linac* or (linear* adj1 accelerator*)).ab,ti. |
| 7 | (cyber knife* or cyberknife*).ab,ti. |
| 8 | radiosurg*.ti,ab. |
| 9 | or/5-8 |
| 10 | and/4,9 |
| 11 | exp cohort studies/ or exp epidemiologic studies/ or exp clinical trial/ or exp evaluation studies as topic/ or exp statistics as topic/ |
| 12 | ((control and (group* or study)) or (time and factors) or program or survey* or ci or cohort or comparative stud* or evaluation studies or follow-up*).mp. |
| 13 | or/11-12 |
| 14 | (animals/ not humans/) or comment/ or editorial/ or exp review/ or meta analysis/ or consensus/ or exp guideline/ |
| 15 | hi.fs. or case report.mp. |
| 16 | or/14-15 |
| 17 | 13 not 16 |
| 18 | (clinical trial* or random* or placebo).ti,ab. |
| 19 | trial.ti. |
| 20 | (search or meta analysis or medline or systematic review).ti,ab. |
| 21 | or/18-20 |
| 22 | 21 not (comment or editorial).pt. |
| 23 | 10 and (17 or 22) |
| 24 | 23 and (english or german).lg. |

Embase via Ovid

Embase 1974 to 2021 September 21

Search filters:

RCTs: Wong [3] – Strategy minimizing difference between sensitivity and specificity

| # | Searches |
| --- | --- |
| 1 | acoustic neuroma/ |
| 2 | "acoustic neurinoma"/ |
| 3 | (vestibular* adj1 schwannoma*).ti,ab. |
| 4 | (acoustic adj1 (neuroma* or tumor*)).ti,ab. |
| 5 | ((cerebellopontine* adj1 angle*) and (tumor* or tumour*)).ti,ab. |
| 6 | or/1-5 |
| 7 | exp radiosurgery/ |
| 8 | gamma knife/ |
| 9 | (gamma* adj1 knife*).ti,ab. |
| 10 | (linac* or (linear* adj1 accelerator*)).mp. |
| 11 | (cyber knife* or cyberknife*).mp. |
| 12 | (stereotactic* adj1 radiosurg*).ti,ab. |
| 13 | or/7-12 |
| 14 | and/6,13 |
| 15 | (random* or double-blind*).tw. |
| 16 | placebo*.mp. |
| 17 | or/15-16 |
| 18 | (meta analysis or systematic review or MEDLINE).tw. |
| 19 | 14 and (17 or 18) |
| 20 | 19 not medline.cr. |
| 21 | 20 not (exp animal/ not exp humans/) |
| 22 | 21 not (Conference Abstract or Conference Review or Editorial).pt. |

The Cochrane Library via Wiley

- Cochrane Database of Systematic Reviews: Issue 9 of 12, September 2021

Cochrane Central Register of Controlled Trials: Issue 9 of 12, September 2021

| ID | Search |
| --- | --- |
| #1 | MeSH descriptor: [Neuroma, Acoustic] this term only |
| #2 | MeSH descriptor: [Cerebellopontine Angle] this term only |
| #3 | (vestibular* near/1 schwannoma*):ti,ab |
| #4 | (acoustic* near/1 (neuroma* or tumor*)):ti,ab |
| #5 | ((cerebellopontine* near/1 angle*) and (tumor* or tumour*)):ti,ab |
| #6 | #1 or #2 OR #3 or #4 or #5 |
| #7 | MeSH descriptor: [Radiosurgery] this term only |
| #8 | (gamma* near/1 knife*):ti,ab |
| #9 | (linac* or (linear* near/1 accelerator*)):ti,ab |
| #10 | (cyber knife* or cyberknife*):ti,ab |
| #11 | (stereotactic* near/1 radiosurg*):ti,ab |
| #12 | #7 or #8 or #9 or #10 or #11 |
| #13 | #6 and #12 |
| #14 | #13 not ((language next (afr or ara or aze or bos or bul or car or cat or chi or cze or dan or dut or es or est or fin or fre or gre or heb or hrv or hun or ice or ira or ita or jpn or ko or kor or lit or nor or peo or per or pol or por or pt or rom or rum or rus or slo or slv or spa or srp or swe or tha or tur or ukr or urd or uzb)) not (language near/2 (en or eng or english or ger or german or mul or unknown))) |
| #15 | #14 not (*clinicaltrial*gov* or *who*trialsearch* or *clinicaltrialsregister*eu* or *anzctr*org*au* or *trialregister*nl* or *irct*ir* or *isrctn* or *controlled*trials*com* or *drks*de*):so |
| #16 | #13 in Cochrane Reviews, Cochrane Protocols |
| #17 | #15 in Trials |

Health Technology Assessment Database via INAHTA

| # | Searches |
| --- | --- |
| 1 | ((schwannoma* OR acoustic OR cerebellopontine*) AND (gamma OR knife OR linac* OR accelerator* OR cyberknife* or radiosurg*)) |

ClinicalTrials.gov

U.S. National Institutes of Health

- URL: <http://www.clinicaltrials.gov>
- Advanced Search

| Searches |
| --- |
| (gamma knife OR cyber knife OR linear accelerator OR stereotactic radiosurgery) AND (brain metastasis OR cerebral metastasis OR cavity resection OR acoustic neuroma) |

International Clinical Trials Registry Platform Search Portal

World Health Organization

- URL: <http://apps.who.int/trialsearch>
- Standard Search

| Searches |
| --- |
| gamma knife OR cyber knife OR cyberknife OR linear accelerator OR linac OR stereotactic (without Synonyms) |

**Manufacturers contacted**

1. Accuray
2. BrainLAB
3. Elekta
4. Gamma Star
5. Neusoft Medical Systems
6. Panacea Medical Technologies
7. Shinva Medical Instrument
8. Siemens
9. Varian
10. Vision RT
11. ZAP Surgical Systems

**References**

1. Lefebvre C, Glanville J, Briscoe S et al. Cochrane Handbook for Systematic Reviews of Interventions Version 6; Technical Supplement to Chapter 4: Searching for and selecting studies [online]. 2019 [Zugriff: 09.04.2021]. URL: <https://training.cochrane.org/handbook/version-6/chapter-4-tech-suppl>.

2. Waffenschmidt S, Navarro-Ruan T, Hobson N et al. Development and validation of study filters for identifying controlled non-randomized studies in PubMed and Ovid MEDLINE. Res Synth Methods 2020; 11(5): 617-626. <https://dx.doi.org/10.1002/jrsm.1425>.

3. Wong SS, Wilczynski NL, Haynes RB. Comparison of top-performing search strategies for detecting clinically sound treatment studies and systematic reviews in MEDLINE and EMBASE. J Med Libr Assoc 2006; 94(4): 451-455.
