# Supplementary material for: Single-fraction stereotactic radiosurgery versus microsurgical resection for the treatment of vestibular schwannoma: a systematic review and meta-analysis
Source: Syst Rev. 2022 Dec 12;11:265. doi: 10.1186/s13643-022-02118-9 (PMC9743510; doi:10.1186/s13643-022-02118-9)
Supplement: Supplementary file 2 — Additional file 2. List of excluded studies. [file 13643_2022_2118_MOESM2_ESM.docx]

**Additional file 2: List of excluded studies**

Ineligible population

1. Kalash R, Glaser SM, Flickinger JC et al. Stereotactic body radiation therapy for benign spine tumors: is dose de-escalation appropriate? Journal of Neurosurgery Spine 2018; 29(2): 220-225. <https://dx.doi.org/10.3171/2017.12.Spine17920>.

Ineligible intervention

1. Pan HC, Sheehan J, Sheu ML et al. Intracapsular decompression or radical resection followed by Gamma Knife surgery for patients harboring a large vestibular schwannoma. J Neurosurg 2012; 117 Suppl: 69-77. <https://dx.doi.org/10.3171/2012.6.Gks12697>.

Ineligible control group

1. Duenas SM, Pun J, Radwan HA et al. A Randomized Trial on the Efficacy of Topical Anesthesia for Pain Reduction during Frame Placement for Gamma Knife Radiosurgery. Stereotact Funct Neurosurg 2016; 94(4): 259-264. <https://dx.doi.org/10.1159/000449013>.

2. Regis J, Tamura M, Guillot C et al. Radiosurgery with the world's first fully robotized leksell gamma knife perfeXion in clinical use: A 200-patient prospective, randomized, controlled comparison with the gamma knife 4C. Neurosurgery 2009; 64(2): 346-355. <https://dx.doi.org/10.1227/01.Neu.0000337578.00814.75>.

3. Yomo S, Tamura M, Carron R et al. A quantitative comparison of radiosurgical treatment parameters in vestibular schwannomas: the Leksell Gamma Knife Perfexion versus Model 4C. Acta Neurochir (Wien) 2010; 152(1): 47-55. <https://dx.doi.org/10.1007/s00701-009-0510-3>.

Ineligible outcome

1. Leon J, Trifiletti DM, Waddle MR et al. Trends in the initial management of vestibular schwannoma in the United States. J Clin Neurosci 2019; 68: 174-178. <https://dx.doi.org/10.1016/j.jocn.2019.07.002>.

Ineligible study design

1. Apicella G, Paolini M, Deantonio L et al. Radiotherapy for vestibular schwannoma: Review of recent literature results. Rep Pract Oncol Radiother 2016; 21(4): 399-406. <https://dx.doi.org/10.1016/j.rpor.2016.02.002>.

2. Buss EJ, Wang TJC, Sisti MB. Stereotactic radiosurgery for management of vestibular schwannoma: a short review. Neurosurg Rev 2020. <https://dx.doi.org/10.1007/s10143-020-01279-2>.

3. Carlson ML, Vivas EX, McCracken DJ et al. Congress of Neurological Surgeons Systematic Review and Evidence-Based Guidelines on Hearing Preservation Outcomes in Patients With Sporadic Vestibular Schwannomas. Neurosurgery 2018; 82(2): E35-E39. <https://dx.doi.org/10.1093/neuros/nyx511>.

4. Chung LK, Nguyen TP, Sheppard JP et al. A Systematic Review of Radiosurgery Versus Surgery for Neurofibromatosis Type 2 Vestibular Schwannomas. World Neurosurg 2018; 109: 47-58. <https://dx.doi.org/10.1016/j.wneu.2017.08.159>.

5. Coughlin AR, Willman TJ, Gubbels SP. Systematic Review of Hearing Preservation After Radiotherapy for Vestibular Schwannoma. Otol Neurotol 2018; 39(3): 273-283. <https://dx.doi.org/10.1097/mao.0000000000001672>.

6. Ding K, Ng E, Romiyo P et al. Meta-analysis of tumor control rates in patients undergoing stereotactic radiosurgery for cystic vestibular schwannomas. Clin Neurol Neurosurg 2020; 188: 105571. <https://dx.doi.org/10.1016/j.clineuro.2019.105571>.

7. Fabbris C, Gazzini L, Paltrinieri D et al. Exclusive surgical treatment for vestibular schwannoma regrowth or recurrence: A meta-analysis of the literature. Clin Neurol Neurosurg 2020; 193: 105769. <https://dx.doi.org/10.1016/j.clineuro.2020.105769>.

8. Galloway L, Palaniappan N, Shone G et al. Trigeminal neuropathy in vestibular schwannoma: a treatment algorithm to avoid long-term morbidity. Acta Neurochir (Wien) 2018; 160(4): 681-688. <https://dx.doi.org/10.1007/s00701-017-3452-1>.

9. Gauden A, Weir P, Hawthorne G et al. Systematic review of quality of life in the management of vestibular schwannoma. J Clin Neurosci 2011; 18(12): 1573-1584. <https://dx.doi.org/10.1016/j.jocn.2011.05.009>.

10. Germano IM, Sheehan J, Parish J et al. Congress of Neurological Surgeons Systematic Review and Evidence-Based Guidelines on the Role of Radiosurgery and Radiation Therapy in the Management of Patients With Vestibular Schwannomas. Neurosurgery 2018; 82(2): E49-E51. <https://dx.doi.org/10.1093/neuros/nyx515>.

11. Hadjipanayis CG, Carlson ML, Link MJ et al. Congress of Neurological Surgeons Systematic Review and Evidence-Based Guidelines on Surgical Resection for the Treatment of Patients With Vestibular Schwannomas. Neurosurgery 2018; 82(2): E40-E43. <https://dx.doi.org/10.1093/neuros/nyx512>.

12. Karpinos M, Teh BS, Zeck O et al. Treatment of acoustic neuroma: stereotactic radiosurgery vs. microsurgery. Int J Radiat Oncol Biol Phys 2002; 54(5): 1410-1421. <https://dx.doi.org/10.1016/s0360-3016(02)03651-9>.

13. Kaylie DM, Horgan MJ, Delashaw JB et al. A meta-analysis comparing outcomes of microsurgery and gamma knife radiosurgery. Laryngoscope 2000; 110(11): 1850-1856. <https://dx.doi.org/10.1097/00005537-200011000-00016>.

14. Kim BS, Seol HJ, Lee JI et al. Clinical outcome of neurofibromatosis type 2-related vestibular schwannoma: treatment strategies and challenges. Neurosurg Rev 2016; 39(4): 643-653. <https://dx.doi.org/10.1007/s10143-016-0728-5>.

15. Mahboubi H, Sahyouni R, Moshtaghi O et al. CyberKnife for Treatment of Vestibular Schwannoma: A Meta-analysis. Otolaryngol Head Neck Surg 2017; 157(1): 7-15. <https://dx.doi.org/10.1177/0194599817695805>.

16. Maniakas A, Saliba I. Microsurgery versus stereotactic radiation for small vestibular schwannomas: a meta-analysis of patients with more than 5 years'follow-up. Otol Neurotol 2012; 33(9): 1611-1620. <https://dx.doi.org/10.1097/MAO.0b013e31826dbd02>.

17. McLaughlin EJ, Bigelow DC, Lee JY et al. Quality of life in acoustic neuroma patients. Otol Neurotol 2015; 36(4): 653-656. <https://dx.doi.org/10.1097/mao.0000000000000674>.

18. Olson JJ, Kalkanis SN, Ryken TC. Congress of Neurological Surgeons Systematic Review and Evidence-Based Guidelines on the Treatment of Adults with Vestibular Schwannomas: Executive Summary. Clin Neurosurg 2018; 82(2): 129-134. <https://dx.doi.org/10.1093/neuros/nyx586>.

19. Papatsoutsos E, Spielmann PM. Self-Evaluated Quality of Life and Functional Outcomes After Microsurgery, Stereotactic Radiation or Observation-Only for Vestibular Schwannoma of the Adult Patient: A Systematic Review. Otol Neurotol 2018; 39(2): 232-241. <https://dx.doi.org/10.1097/mao.0000000000001664>.

20. Peng KA, Wilkinson EP. Optimal outcomes for hearing preservation in the management of small vestibular schwannomas. J Laryngol Otol 2016; 130(7): 606-610. <https://dx.doi.org/10.1017/s0022215116007969>.

21. Pollock BE. Vestibular schwannoma management: an evidence-based comparison of stereotactic radiosurgery and microsurgical resection. Prog Neurol Surg 2008; 21: 222-227. <https://dx.doi.org/10.1159/000157170>.

22. Pollock BE, Lunsford LD, Kondziolka D et al. Outcome analysis of acoustic neuroma management: a comparison of microsurgery and stereotactic radiosurgery. Neurosurgery 1995; 36(1): 215-224; discussion 224-219. <https://dx.doi.org/10.1227/00006123-199501000-00036>.

23. Regis J, Pellet W, Delsanti C et al. Functional outcome after gamma knife surgery or microsurgery for vestibular schwannomas. J Neurosurg 2002; 97(5): 1091-1100. <https://dx.doi.org/10.3171/jns.2002.97.5.1091>.

24. Romiyo P, Ng E, Dejam D et al. Radiosurgery treatment is associated with improved facial nerve preservation versus repeat resection in recurrent vestibular schwannomas. Acta Neurochir (Wien) 2019; 161(7): 1449-1456. <https://dx.doi.org/10.1007/s00701-019-03940-2>.

25. Tsao MN, Sahgal A, Xu W et al. Stereotactic radiosurgery for vestibular schwannoma: International Stereotactic Radiosurgery Society (ISRS) Practice Guideline. Journal of Radiosurgery and SBRT 2017; 5(1): 5-24.

26. Whitmore RG, Urban C, Church E et al. Decision analysis of treatment options for vestibular schwannoma. J Neurosurg 2011; 114(2): 400-413. <https://dx.doi.org/10.3171/2010.3.Jns091802>.

27. Yamakami I, Uchino Y, Kobayashi E et al. Conservative management, gamma-knife radiosurgery, and microsurgery for acoustic neurinomas: a systematic reviewof outcome and risk of three therapeutic options. Neurol Res 2003; 25(7): 682-690. <https://dx.doi.org/10.1179/016164103101202075>.
