# Supplementary material for: Single-fraction stereotactic radiosurgery versus microsurgical resection for the treatment of vestibular schwannoma: a systematic review and meta-analysis
Source: Syst Rev. 2022 Dec 12;11:265. doi: 10.1186/s13643-022-02118-9 (PMC9743510; doi:10.1186/s13643-022-02118-9)
Supplement: Supplementary file 3 — Additional file 3. Details on patient characteristics. [file 13643_2022_2118_MOESM3_ESM.docx]

**Additional file 3: Details on patient characteristics**

| Table 1: Patient characteristics at baseline (Carlson 2021) | | |
| --- | --- | --- |
| Characteristics  Category | sfSRS | MR |
| N | 48 | 118 |
| age [years], mean (SD) | 61 (10) | 52 (14) |
| sex [female / male], % | 46 / 54 | 48 / 52 |
| tumour size [cm], n (%) |  |  |
| 0–0.9 | 19 (40) | 23 (19) |
| 1–1.9 | 23 (48) | 45 (38) |
| 2–2.9 | 6 (13) | 34 (29) |
| 3–3.9 | 0 (0) | 12 (10) |
| ≥ 4 | 0 (0) | 4 (3) |
| tumour location, n (%) |  |  |
| cerebellopontine angle | 39 (81) | 97 (82) |
| intracanalicular | 9 (19) | 21 (18) |
| hearing function [AAO-HNS classification], n (%) |  |  |
| Grade A | 10 (21) | 31 (27) |
| Grade B | 21 (45) | 32 (28) |
| Grade C | 3 (6) | 14 (12) |
| Grade D | 13 (28) | 37 (32) |
| normal facial function [HB‑Grad 1], n (%) | 47 (98) | 114 (97) |
| AAO-HNS: American Academy of Otolaryngology-Head and Neck Surgery; HB: House-Brackmann; MR: microsurgical resection; N: number of included patients; n: number of patients in the respective category; SD: standard deviation; sfSRS: single-fraction stereotactic radiosurgery | | |

| Table 2: Patient characteristics at baseline (Myrseth 2009) | | |
| --- | --- | --- |
| Characteristics  Category | sfSRS | MR |
| N | 60 | 28 |
| age [years], mean [min.; max.] | 57.5 [36; 79] | 52.5 [26; 73] |
| sex [female / male], %^a^ | 56.7 / 43.3 | 57.1 / 42.9 |
| hearing function [Gardner-Robertson classification], n (%) |  |  |
| A oder B | 25 (42.3) | 13 (44.4) |
| normal facial function [HB-Grad 1], n (%) | 60 (100) | 28 (100) |
| vertigo, n (%) | 29 (48.3) | 13 (46.4) |
| tinnitus, n (%) | 49 (81.7) | 24 (85.7) |
| balance function, n (%) | 22 (36.7) | 12 (42.9) |
| tumour size [cm], mean (SD) | 1.6 (n. r.) | 1.8 (n. r.) |
| a. IQWiG’s own calculation | | |
| HB: House-Brackmann; MR microsurgical resection; N: number of included patients; n: number of patients in the respective category; n. r.: not reported; SD: standard deviation; sfSRS: single-fraction stereotactic radiosurgery | | |

| Table 3: Patient characteristics at baseline (Pollock 2006) | | |
| --- | --- | --- |
| Characteristics  Category | sfSRS | MR |
| N | 46 | 36 |
| age [years], mean (SD) | 53.9 (n. r.) | 48.2 (n. r.) |
| sex [female / male], %^a^ | 41.3 / 58.7 | 47.2 / 52.8 |
| facial weakness, n (%) | 0 (0) | 0 (0) |
| facial numbness, n (%) | 1 (2) | 2 (6) |
| hearing function [AAO-HNS Grade A or B], n (%) | 30 (65) | 22 (61) |
| vertigo [DHI]^b^, mean (SD) | 11.0 (n. r.) | 11.9 (n. r.) |
| tinnitus [VAS]^b^, mean (SD) | 9.0 (n. r.) | 11.6 (n. r.) |
| headache [score]^c^, mean (SD) | 6.0 (n. r.) | 5.7 (n. r.) |
| intracanalicular tumour, n (%) | 10 (22) | 5 (14) |
| tumour size [cm], mean (SD) | 1.23 (n. r.) | 1.41 (n. r.) |
| a. IQWiG’s own calculation  b. range of 0 to100, higher values corresponding to greater perceived symptom  c. range of 0 to 20, higher values corresponding to greater perceived symptom | | |
| AAO-HNS: American Academy of Otolaryngology-Head and Neck Surgery; DHI: Dizziness Handicap Inventory; MR microsurgical resection; N: number of included patients; n: number of patients in the respective category; n. r.: not reported; SD: standard deviation; sfSRS: single-fraction stereotactic radiosurgery; VAS: visual analogue scale | | |
