# Supplementary material for: Single-fraction stereotactic radiosurgery versus microsurgical resection for the treatment of vestibular schwannoma: a systematic review and meta-analysis
Source: Syst Rev. 2022 Dec 12;11:265. doi: 10.1186/s13643-022-02118-9 (PMC9743510; doi:10.1186/s13643-022-02118-9)
Supplement: Supplementary file 4 — Additional file 4. Detailed results not presented in the manuscript. [file 13643_2022_2118_MOESM4_ESM.docx]

**Additional file 4: Detailed results not presented in the manuscript**

**List of tables**

[Table 1: Results – hearing function (dichotomous) 1](#_Toc80187499)

[Table 2: Results – hearing function (continuous) 2](#_Toc80187500)

[Table 3: Results – headache 3](#_Toc80187501)

[Table 4: Results – balance function 3](#_Toc80187502)

[Table 5: Results – work disability 4](#_Toc80187503)

[Table 6: Results – health-related quality of life (PANQOL) 4](#_Toc80187504)

[Table 7: Results – health-related quality of life (GBI) 6](#_Toc80187505)

[Table 8: Results – health-related quality of life (Tinnitus Survey) 7](#_Toc80187506)

[Table 9: Results – health-related quality of life (SF-36) 8](#_Toc80187507)

| Table 1: Results – hearing function (dichotomous) | | | | | | | | | | |
| --- | --- | --- | --- | --- | --- | --- | --- | --- | --- | --- |
| Study  Instrument  Grade | Time of evaluation | sfSRS | | |  | MR | | |  | sfSRS vs. MR |
|  |  | N | n | % |  | N | n | % |  | OR [95 %‑CI]; p‑value |
| Myrseth 2009 |  |  |  |  |  |  |  |  |  |  |
| Gardner-Robertson scale | |  |  |  |  |  |  |  |  |  |
| A or B^a^ | Baseline | 59 | 25^b^ | 42.4^b^ |  | 28 | 13^b^ | 46.4^b^ |  | – |
|  | 12 months | 56 | 19^b^ | 33.9^b^ |  | 28 | 0 | 0^d^ |  | 29.64 [1.72; 511.93]^c^; < 0.001^d^ |
|  | 24 months | 60 | 17^b^ | 28.3^b^ |  | 28 | 0 | 0^d^ |  | 22.93 [1.33; 396.64]^c^; 0.002^d^ |
| Pollock 2006 |  |  |  |  |  |  |  |  |  |  |
| AAO-HNS |  |  |  |  |  |  |  |  |  |  |
| A or B^a^ | Baseline | 46 | 30 | 65 |  | 36 | 22 | 61 |  | – |
|  | 3 months | n. r. | n. r. | 77 |  | n. r. | n. r. | 5 |  | n. r.; < 0.001^e^ |
|  | 12 months | n. r. | n. r. | 63 |  | n. r. | n. r. | 5 |  | n. r.; < 0.001^e^ |
|  | Last follow-up^f^ | n. r. | n. r. | 63 |  | n. r. | n. r. | 5 |  | n. r.; < 0.001^e^ |
| a. considered functionally preserved  b. IQWiG’s own calculation  c. IQWiG’s own calculation (asymptotic)  d. IQWiG’s own calculation (unconditional exact test, CSZ method according to [1])  e. statistical test not reported  f. mean follow-up was 42 months (range of 12 to 62 months). | | | | | | | | | | |
| AAO-HNS: American Academy of Otolaryngology-Head and Neck Surgery; CI: confidence interval; MR: microsurgical resection; n: number of patients with an event; N: number of analysed patients; n. r.: not reported; OR: Odds Ratio; sfSRS: single-fraction stereotactic radiosurgery; vs.: versus | | | | | | | | | | |

| Table 2: Results – hearing function (continuous) | | | | | | | | |
| --- | --- | --- | --- | --- | --- | --- | --- | --- |
| Study  Instrument (range)  Time of evaluation  Intervention  Comparison | N^a^ | Values at baseline | | Changes compared with baseline | | sfSRS vs. MR | | |
|  |  | Mean^b^ | SD^b^ | Mean^b^ | [95 %-CI]^b^ | MD^c^ | [95 %-CI]^c^ | p-value^c^ |
| Carlson 2021 |  |  |  |  |  |  |  |  |
| Likert scale (range of 1 [normal hearing] to 10 [completely deaf]) | | | | | | | | |
| Last follow-up^d^ | | | | | | | | |
| sfSRS | 48 | 5.9 | 2.5 | 1.3 | [0.5; 2.2] | −1.60 | [−2.63; −0.57] | 0.002 |
| MR | 118 | 5.9 | 2.8 | 2.9 | [2.3; 3.4] |  |  |  |
| a. number of patients included in the evaluation to calculate the effect estimate; values at baseline may be based on other patient numbers  b. adjusted for age, sex, years between study entry and last survey, and tumour size at study entry, tumour location, and hearing class  c. IQWiG’s own calculation (t-Test)  d. mean follow-up was 2.1 years (SD: 1.2 years). | | | | | | | | |
| CI: confidence interval; MD: mean difference; MR: microsurgical resection; N: number of analysed patients; SD: standard deviation; sfSRS: single-fraction stereotactic radiosurgery; vs.: versus | | | | | | | | |

| Table 3: Results – headache | | | | | | | | | | | |
| --- | --- | --- | --- | --- | --- | --- | --- | --- | --- | --- | --- |
| Study  Instrument (range)  Time of evaluation  Intervention  Comparison | | N^a^ | Values at baseline | | | Values at time of evaluation | | | sfSRS vs. MR | | |
|  |  |  | Mean | SD | | Mean | | SD | MD | [95 %-CI] | p-value |
| Carlson 2021 |  | |  |  | |  | |  |  |  |  |
| Likert scale (range of 1 to 10, higher scores correspond to greater perceived symptom) | | | | | | | | | | | |
| Last follow-up^b^ | | | | | | | | | | | |
| sfSRS | 48 | | 2.1^c^ | 1.9^c^ | | 0.2^c, d^ | | [−0.8; 1.2]^c, e^ | −0.10^f^ | [−1.31; 1.11]^f^ | 0.871^f^ |
| MR | 118 | | 3.4^c^ | 2.6^c^ | | 0.3^c, d^ | | [−0.4; 0.9]^c, e^ |  |  |  |
| Pollock 2006 | |  |  | |  |  |  | |  |  |  |
| Headache Survey (range of 0 to 20, higher scores correspond to greater perceived symptom) | | | | | | | | | | | |
| 3 months | |  |  |  | |  | |  |  |  |  |
| sfSRS | | 46 | 6.0 | n. r. | | 5.7 | | n. r. | n. r. | n. r. | 0.93^g^ |
| MR | | 36 | 5.7 | n. r. | | 5.6 | | n. r. |  |  |  |
| 12 months | |  |  |  | |  | |  |  |  |  |
| sfSRS | | 46 | 6.0 | n. r. | | 5.2 | | n. r. | n. r. | n. r. | 0.18^g^ |
| MR | | 36 | 5.7 | n. r. | | 6.7 | | n. r. |  |  |  |
| Last follow-up^h^ | |  |  |  | |  | |  |  |  |  |
| sfSRS | | 46 | 6.0 | n. r. | | 5.0 | | n. r. | n. r. | n. r. | 0.29^g^ |
| MR | | 36 | 5.7 | n. r. | | 6.0 | | n. r. |  |  |  |
| a. number of patients included in the evaluation to calculate the effect estimate; values at baseline may be based on other patient numbers  b. mean follow-up was 2.1 years (SD: 1.2 years).  c. adjusted for age, sex, years between study entry and last survey, and tumour size at study entry, tumour location, and hearing class  d. changes compared with baseline  e. 95 %-CI  f. IQWiG’s own calculation (t-Test)  g. statistical test not reported  h. mean follow-up was 42 months (range of 12 to 62 months). | | | | | | | | | | | |
| CI: confidence interval; MD: mean difference; MR: microsurgical resection; N: number of analysed patients; n r.: not reported; SD: standard deviation; sfSRS: single-fraction stereotactic radiosurgery; vs.: versus | | | | | | | | | | | |

| Table 4: Results – balance function | | | | | | | | | | |
| --- | --- | --- | --- | --- | --- | --- | --- | --- | --- | --- |
| Study | Time of evaluation | sfSRS | | |  | MR | | |  | sfSRS vs. MR |
|  |  | N | n | % |  | N | n | % |  | OR [95 %‑CI]; p‑value |
| Myrseth 2009 | Baseline | n. r. | n. r. | 36.7 |  | n. r. | n. r. | 42.9 |  | – |
|  | 12 months | n. r. | n. r. | 38.6 |  | n. r. | n. r. | 39.3 |  | n. r.; n. s. |
|  | 24 months | n. r. | n. r. | 45.0 |  | n. r. | n. r. | 50.0 |  | n. r.; n. s. |
| CI: confidence interval; MR: microsurgical resection; n: number of patients with an event; N: number of analysed patients; n. r.: not reported; n. s.: not significant; OR: Odds Ratio; sfSRS: single-fraction stereotactic radiosurgery; vs.: versus | | | | | | | | | | |

| Table 5: Results – work disability | | | | | | | | | |
| --- | --- | --- | --- | --- | --- | --- | --- | --- | --- |
| Study  Time of evaluation  Status | sfSRS | | |  | MR | | |  | sfSRS vs. MR |
|  | N^a^ | n | %^a^ |  | N^a^ | n | %^a^ |  | p‑value |
| Myrseth 2009 |  |  |  |  |  |  |  |  |  |
| baseline |  |  |  |  |  |  |  |  |  |
| working | 60 | 29 | 48.3 |  | 28 | 11 | 39.3 |  | – |
| sick leave | 60 | 11 | 18.3 |  | 28 | 9 | 32.1 |  |  |
| disability | 60 | 7 | 11.7 |  | 28 | 2 | 7.1 |  |  |
| retired | 60 | 13 | 21.7 |  | 28 | 6 | 21.4 |  |  |
| 24 months |  |  |  |  |  |  |  |  |  |
| working | 60 | 27 | 45.0 |  | 28 | 14 | 50.0 |  | 0.924^b^ |
| sick leave | 60 | 5 | 8.3 |  | 28 | 3 | 10.7 |  |  |
| disability | 60 | 12 | 20.0 |  | 28 | 5 | 17.9 |  |  |
| retired | 60 | 16 | 26.7 |  | 28 | 6 | 21.4 |  |  |
| a. IQWiG’s own calculation  b. IQWiG’s own calculation (χ^2^-Test) | | | | | | | | | |
| MR: microsurgical resection; n: number of patients with an event; N: number of analysed patients; sfSRS: single-fraction stereotactic radiosurgery; vs.: versus | | | | | | | | | |

| Table 6: Results – health-related quality of life (PANQOL)  (multi-page table) | | | | | | | | | | |
| --- | --- | --- | --- | --- | --- | --- | --- | --- | --- | --- |
| Study  Instrument (range)  Time of evaluation  Intervention  Comparison | N^a^ | Values at baseline | | Values at time of evaluation | | Changes compared with baseline | | sfSRS vs. MR | | |
|  |  | Mean^b^ | [95 %-CI]^b^ | Mean^b^ | [95 %-CI]^b^ | Mean^b^ | [95 %-CI]^b^ | MD^c^ | [95 %-CI]^c^ | p-value^c^ |
| Carlson 2021  PANQOL |  |  |  |  |  |  |  |  |  |  |
| Total score (range of 0 to 100, higher values correspond to lower perceived symptom) | | | | | | | | | | |
| Last follow-up^d^ | | | | | | | | | | |
| sfSRS | 48 | 70 | [64; 76] | 70 | [63; 77] | 0.3 | [−5.3; 5.9] | 5.00 | [−3.41; 13.41] | 0.242 |
| MR | 118 | 65 | [61; 69] | 65 | [60; 69] | −0.1 | [−3.8; 3.5] |  |  |  |
| Domain score anxiety (range of 0 to 100, higher values correspond to lower perceived symptom) | | | | | | | | | | |
| Last follow-up^d^ | | | | | | | | | | |
| sfSRS | 48 | 73 | [64; 81] | 78 | [68; 88] | 5.3 | [−4.9; 15.5] | 2.00 | [−10.11; 14.11] | 0.745 |
| MR | 118 | 65 | [59; 71] | 76 | [69; 82] | 10.8 | [4.1; 17.5] |  |  |  |
| Domain score facial function (range of 0 to 100, higher values correspond to lower perceived symptom) | | | | | | | | | | |
| Last follow-up^d^ | | | | | | | | | | |
| sfSRS | 48 | 87 | [81; 93] | 87 | [79; 94] | −0.1 | [−9.0; 8.8] | 9.00 | [0.41; 17.59] | 0.040 |
| MR | 118 | 82 | [78; 86] | 78 | [73; 82] | −4.2 | [−10.1; 1.6] |  |  |  |
| Domain score general health (range of 0 to 100, higher values correspond to lower perceived symptom) | | | | | | | | | | |
| Last follow-up^d^ | | | | | | | | | | |
| sfSRS | 48 | 60 | [53; 67] | 63 | [55; 71] | 3.1 | [−3.6; 9.9] | −3.00 | [−12.42; 6.42] | 0.530 |
| MR | 118 | 65 | [61; 70] | 66 | [61; 71] | 0.5 | [−4.0; 4.9] |  |  |  |
| Domain score balance (range of 0 to 100, higher values correspond to lower perceived symptom) | | | | | | | | | | |
| Last follow-up^d^ | | | | | | | | | | |
| sfSRS | 48 | 70 | [60; 80] | 65 | [56; 75] | −4.8 | [−13.0; 3.4] | 5.00 | [−6.27; 16.27] | 0.382 |
| MR | 118 | 62 | [56; 69] | 60 | [54; 66] | −2.5 | [−7.8; 2.9] |  |  |  |
| Domain score hearing loss (range of 0 to 100, higher values correspond to lower perceived symptom) | | | | | | | | | | |
| Last follow-up^d^ | | | | | | | | | | |
| sfSRS | 48 | 61 | [53; 70] | 57 | [48; 66] | −4.5 | [−12.2; 3.2] | 3.00 | [−8.10; 14.10] | 0.594 |
| MR | 118 | 59 | [54; 65] | 54 | [48; 60] | −4.8 | [−9.9; 0.2] |  |  |  |
| Domain score energy (range of 0 to 100, higher values correspond to lower perceived symptom) | | | | | | | | | | |
| Last follow-up^d^ | | | | | | | | | | |
| sfSRS | 48 | 67 | [57; 77] | 62 | [52; 72] | −5.0 | [−13.5; 3.4] | 3.00 | [−9.11; 15.11] | 0.625 |
| MR | 118 | 60 | [53; 66] | 59 | [52; 65] | −0.9 | [−6.4; 4.7] |  |  |  |
| Domain score pain (range of 0 to 100, higher values correspond to lower perceived symptom) | | | | | | | | | | |
| Last follow-up^d^ | | | | | | | | | | |
| sfSRS | 48 | 72 | [60; 83] | 80 | [69; 91] | 8.4 | [−3.7; 20.4] | 20.00 | [6.20; 33.80] | 0.005 |
| MR | 118 | 60 | [52; 67] | 60 | [52; 67] | 0.1 | [−7.8; 8.0] |  |  |  |
| a. number of patients included in the evaluation to calculate the effect estimate; values at baseline may be based on other patient numbers  b. adjusted for age, sex, years between study entry and last survey, and tumour size at study entry, tumour location, and hearing class  c. IQWiG’s own calculation (t-Test) based on the values at the time of evaluation  d. mean follow-up was 2.1 years (SD: 1.2 years). | | | | | | | | | | |
| CI: confidence interval; MD: mean Difference; MR: microsurgical resection; N: number of analysed patients; PANQOL: Penn Acoustic Neuroma Quality of Life; sfSRS: single-fraction stereotactic radiosurgery; vs.: versus | | | | | | | | | | |

| Table 7: Results – health-related quality of life (GBI) | | | | | | | | | | |
| --- | --- | --- | --- | --- | --- | --- | --- | --- | --- | --- |
| Study  Instrument (range)  Time of evaluation  Intervention  Comparison | N^a^ | Values at baseline | | Values at time of evaluation | | Changes compared with baseline | | sfSRS vs. MR | | |
|  |  | Mean | SD | Mean | SD | Mean | SD | MD^b^ | [95 %-CI]^b^ | p-value^b^ |
| Myrseth 2009  GBI |  |  |  |  |  |  |  |  |  |  |
| Total score (range of −100 to 100, higher values correspond to lower perceived symptom) | | | | | | | | | | |
| 12 months | | | | | | | | | | |
| sfSRS | 55 | −15.1 | 13.7 | −2.5 | 23.6 | n. r. | n. r. | 2.60 | [−9.02; 14.22] | 0.657 |
| MR | 28 | −16.7 | 15.1 | −5.1 | 28.0 | n. r. | n. r. |  |  |  |
| 24 months | | | | | | | | | | |
| sfSRS | 57 | −15.1 | 13.7 | 3.2 | 22.6 | n. r. | n. r. | 13.90 | [3.02; 24.78] | 0.013 |
| MR | 26 | −16.7 | 15.1 | −10.7 | 24.2 | n. r. | n. r. |  |  |  |
| Domain score general (range of −100 to 100, higher values correspond to lower perceived symptom) | | | | | | | | | | |
| 12 months | | | | | | | | | | |
| sfSRS | 55 | −26.9 | 19.1 | −7.3 | 31.2 | n. r. | n. r. | 4.60 | [−10.81; 20.01] | 0.554 |
| MR | 28 | −30.8 | 19.4 | −11.9 | 37.3 | n. r. | n. r. |  |  |  |
| 24 months | | | | | | | | | | |
| sfSRS | 57 | −26.9 | 19.1 | −0.3 | 29.8 | n. r. | n. r. | 16.90 | [2.69; 31.11] | 0.020 |
| MR | 26 | −30.8 | 19.4 | −17.2 | 31.0 | n. r. | n. r. |  |  |  |
| Domain score social (range of −100 to 100, higher values correspond to lower perceived symptom) | | | | | | | | | | |
| 12 months | | | | | | | | | | |
| sfSRS | 55 | 31.6 | 27.6 | 14.5 | 24.9 | n. r. | n. r. | −8.10 | [−19.85; 3.65] | 0.174 |
| MR | 28 | 32.1 | 36.1 | 22.6 | 26.5 | n. r. | n. r. |  |  |  |
| 24 months | | | | | | | | | | |
| sfSRS | 57 | 31.6 | 27.6 | 13.4 | 13.8 | n. r. | n. r. | 0.10 | [−8.38; 8.58] | 0.981 |
| MR | 26 | 32.1 | 36.1 | 13.3 | 25.0 | n. r. | n. r. |  |  |  |
| Domain score physical (range of −100 to 100, higher values correspond to lower perceived symptom) | | | | | | | | | | |
| 12 months | | | | | | | | | | |
| sfSRS | 55 | −13.3 | 14.6 | −1.2 | 22.0 | n. r. | n. r. | 4.80 | [−5.55; 15.15] | 0.359 |
| MR | 28 | −9.6 | 12.6 | −6 | 23.2 | n. r. | n. r. |  |  |  |
| 24 months | | | | | | | | | | |
| sfSRS | 57 | −13.3 | 14.6 | 5.3 | 21.4 | n. r. | n. r. | 15.30 | [4.75; 25.85] | 0.005 |
| MR | 26 | −9.6 | 12.6 | −10.0 | 24.5 | n. r. | n. r. |  |  |  |
| a. number of patients included in the evaluation to calculate the effect estimate; values at baseline may be based on other patient numbers  b. IQWiG’s own calculation (t-Test) | | | | | | | | | | |
| CI: confidence interval; GBI: Glasgow Benefit Inventory questionnaires; MD: mean difference; MR: microsurgical resection ; N: number of analysed patients; n. r.: not reported; SD: standard deviation; sfSRS: single-fraction stereotactic radiosurgery; vs.: versus | | | | | | | | | | |

| Table 8: Results – health-related quality of life (Tinnitus Survey) | | | | | | | | | | |
| --- | --- | --- | --- | --- | --- | --- | --- | --- | --- | --- |
| Study  Instrument (range)  Time of evaluation  Intervention  Comparison | N^a^ | Values at baseline | | Values at time of evaluation | | Changes compared with baseline | | sfSRS vs. MR | | |
|  |  | Mean | SD | Mean | SD | Mean | SD | MD | [95 %-CI] | p-value |
| Pollock 2006 |  |  |  |  |  |  |  |  |  |  |
| Tinnitus Survey (range of 0 to 100, higher values correspond to greater perceived symptom) | | | | | | | | | | |
| 3 months | | | | | | | | | | |
| sfSRS | 46 | 9.0 | n. r. | 10.9 | n. r. | n. r. | n. r. | n. r. | n. r. | 0.51^b^ |
| MR | 36 | 11.6 | n. r. | 10.4 | n. r. | n. r. | n. r. |  |  |  |
| 12 months | | | | | | | | | | |
| sfSRS | 46 | 9.0 | n. r. | 10.3 | n. r. | n. r. | n. r. | n. r. | n. r. | 0.59^b^ |
| MR | 36 | 11.6 | n. r. | 10.7 | n. r. | n. r. | n. r. |  |  |  |
| Last follow-up^c^ | | | | | | | | | | |
| sfSRS | 46 | 9.0 | n. r. | 10.0 | n. r. | n. r. | n. r. | n. r. | n. r. | 0.29^b^ |
| MR | 36 | 11.6 | n. r. | 11.6 | n. r. | n. r. | n. r. |  |  |  |
| a. number of patients included in the evaluation to calculate the effect estimate; values at baseline may be based on other patient numbers  b. statistical test not reported  c. mean follow-up was 42 months (range of 12 to 62 months). | | | | | | | | | | |
| CI: confidence interval; MD: mean difference; MR: microsurgical resection; N: number of analysed patients; n. r.: not reported; SD: standard deviation; sfSRS: single-fraction stereotactic radiosurgery; vs.: versus | | | | | | | | | | |

| Table 9: Results – health-related quality of life (SF-36) | | | | | | | | | | |
| --- | --- | --- | --- | --- | --- | --- | --- | --- | --- | --- |
| Study  Instrument (range)  Time of evaluation  Intervention  Comparison | N^a^ | Values at baseline | | Values at time of evaluation | | Changes compared with baseline | | sfSRS vs. MR | | |
|  |  | Mean | SD | Mean | SD | Mean | SD | MD^b^ | [95 %-CI]^b^ | p-value^b^ |
| Pollock 2006 | | | | | | | | | | |
| SF-36 Mental Component Summary (MCS) (range of 0 to 100, higher values correspond to lower perceived symptom) | | | | | | | | | | |
| 3 months | | | | | | | | | | |
| sfSRS | 42 | 50.4 | 10.1 | 53.3 | 8.9 | 2.0 | 6.0 | 3.00 | [−1.21; 7.21] | 0.159 |
| MR | 35 | 47.9 | 10.7 | 50.3 | 9.6 | 2.7 | 10.1 |  |  |  |
| 12 months | | | | | | | | | | |
| sfSRS | 44 | 50.4 | 10.1 | 54.2 | 7.2 | 2.5 | 7.8 | 2.20 | [−1.06; 5.46] | 0.183 |
| MR | 36 | 47.9 | 10.7 | 52.0 | 7.4 | 4.0 | 9.6 |  |  |  |
| Last follow-up^c^ | | | | | | | | | | |
| sfSRS | 39 | 50.4 | 10.1 | 54.9 | 7.4 | 2.7 | 7.9 | 3.30 | [−0.41; 7.01] | 0.080 |
| MR | 31 | 47.9 | 10.7 | 51.6 | 8.1 | 3.5 | 10.4 |  |  |  |
| SF-36 Physical Component Summary (PCS) (range of 0 to 100, higher values correspond to lower perceived symptom) | | | | | | | | | | |
| 3 months | | | | | | | | | | |
| sfSRS | 42 | 51.6 | 8.4 | 49.5 | 9.0 | −2.7 | 5.0 | 5.70 | [1.68; 9.72] | 0.006 |
| MR | 35 | 52.8 | 7.8 | 43.8 | 8.6 | −8.8 | 12.0 |  |  |  |
| 12 months | | | | | | | | | | |
| sfSRS | 44 | 51.6 | 8.4 | 49.3 | 9.3 | −2.6 | 6.8 | 1.80 | [−2.30; 5.90] | 0.385 |
| MR | 36 | 52.8 | 7.8 | 47.5 | 9.0 | −5.2 | 10.8 |  |  |  |
| Last follow-up^c^ | | | | | | | | | | |
| sfSRS | 39 | 51.6 | 8.4 | 48.5 | 10.7 | −3.4 | 9.3 | −0.70 | [−5.35; 3.95] | 0.765 |
| MR | 31 | 52.8 | 7.8 | 49.2 | 8.2 | −3.7 | 8.8 |  |  |  |
| a. number of patients included in the evaluation to calculate the effect estimate; values at baseline may be based on other patient numbers  b. IQWiG’s own calculation (t-Test) based on the values at the time of evaluation  c. mean follow-up was 42 months (range of 12 to 62 months). | | | | | | | | | | |
| CI: confidence interval; MD: mean difference; MR: microsurgical resection ; N: number of analysed patients; n. r.: not reported; SD: standard deviation; sfSRS: single-fraction stereotactic radiosurgery; SF-36: Short Form-36 Health Survey; vs.: versus | | | | | | | | | | |

**References**

1. Martín Andrés A, Silva Mato A. Choosing the optimal unconditioned test for comparing two independent proportions. Computat Stat Data Anal 1994; 17(5): 555-574. <https://dx.doi.org/10.1016/0167-9473(94)90148-1>.
