# Supplementary material for: Single-fraction stereotactic radiosurgery versus microsurgical resection for the treatment of vestibular schwannoma: a systematic review and meta-analysis
Source: Syst Rev. 2022 Dec 12;11:265. doi: 10.1186/s13643-022-02118-9 (PMC9743510; doi:10.1186/s13643-022-02118-9)
Supplement: Supplementary file 5 — Additional file 5. Overview of key findings according to GRADE. [file 13643_2022_2118_MOESM5_ESM.docx]

#### Additional file 5: Overview of key findings according to GRADE

| **Summary of findings: sfSRS versus MR in patients with vestibular schwannoma** | | | | | | | | | | |
| --- | --- | --- | --- | --- | --- | --- | --- | --- | --- | --- |
| **Patient or population**: patients with vestibular schwannoma  **Setting**: Any | | | **Intervention**: sfSRS  **Comparison**: MR | | | | | | | |
| **Outcomes** | **Anticipated absolute effects^*^** (95% CI) | | | **Relative effect (95% CI)** | **№ of participants  (studies)** | | **Certainty of the evidence (GRADE)** | | **Comments** | |
|  | **Risk with MR** | **Risk with sfSRS** | |  |  |  |  |  |  |  |
| **Mortality** | Information regarding mortality was available descriptively in one study (Myrseth 2009, 91 participants). No deaths occurred during the study period of 2 years. | | | | | | | – | | The evidence on the effect of sfSRS on mortality is very uncertain. |
| **Facial palsy**  Scale used:   1. House-Brackmann score^a^ | 36 per 100 | **3 per 100** (1 to 10) | | **OR 0.06** (0.02 to 0.21) | | 173 (2 studies) | | ⊕⊕⊝⊝ LOW ^1,2^ | | The odds of suffering facial palsy was about 17 times lower when treated with sfSRS compared with treatment with MR.  The evidence suggests sfSRS results in a reduction in facial palsy. |
| **Hearing function**  Scale used:   1. Likert scale^b^ 2. Gardner-Robertson scale^c^ 3. AAO-HNS classification^c^ | **Carlson 2021** (Likert scale): There was a statistically significant difference at the 2.1-year time point (mean) in favour of sfSRS compared with MR (MD: −1.60, 95% CI: [−2.63, −0.57], p = 0.002)  **Myrseth 2009** (Gardner-Robertson scale): The results showed a statistically significant difference in favour of sfSRS compared with MR at the 24-month time point (OR: 22.93, 95% CI: [1.33, 396.64], p = 0.002).  **Pollock 2006** (AAO-HNS classification): The results showed a statistically significant difference at 42 months (mean) in favour of sfSRS compared with MR (p < 0.001, no further information available) | | | | | | | ⊕⊕⊝⊝ LOW ^1,2^ | | It was not possible to pool these data due to the way they were reported.  Myrseth 2009: The odds of preserving functional hearing was about 23 times higher with treatment with sfSRS compared with treatment with MR.  The evidence suggests sfSRS results in an improved preservation of hearing function. |
| **Serious adverse events** | – | **–** | | **–** | | – | | – | | No study reported this outcome. |
| **Length of hospital stay** | **Myrseth 2009:** There was a statistically significant effect in favour of sfSRS compared with MR (mean [min; max]: 2.5 [2; 5] days compared to 12.5 [10; 30] days, p < 0.001).  **Pollock 2006:** sfSRS was performed as an outpatient procedure. The average length of hospital stay after MR was 5.1 days (no further information available). | | | | | | | ⊕⊕⊝⊝ LOW ^1,2^ | | It was not possible to pool these data due to the way they were reported.  The evidence suggests sfSRS results in a reduction in length of hospital stay. |
| **Health-related quality of life**  Scale used:   1. PANQOL^d^ 2. GBI^e^ 3. Tinnitus Survey^f^ 4. SF 36^g^ | **Carlson 2021** (PANQOL): After 2.1 years (mean), the total score showed a numerical advantage of sfSRS compared with MR, but no statistically significant difference between groups (MD: 5.00; 95 % CI: [−3.41, 13.41], p = 0.242).  **Myrseth 2009** (GBI): After 2 years, the total score showed a statistically significant effect in favour of sfSRS compared with MR (MD: 13.90, 95 % CI: [3.02, 24.78], p = 0.013). However, a mean difference of about 14 on a scale of −100 to 100 is not of a magnitude that cannot be explained by bias alone (no dramatic effect).  **Pollock 2006** (Tinnitus Survey) After 42 months (mean), there was no statistically significant difference between groups (p = 0.29).  **Pollock 2006** (The SF 36): After 42 months (mean), the MCS showed a numerical advantage of sfSRS (MD: 3.30, 95 % CI: [−0.41, 7.01], p = 0.080), and the PCS showed a numerical disadvantage of sfSRS (MD: –0.70, 95% CI: [−5.35, 3.95], p = 0.765). However, there was no statistically significant difference between groups. | | | | | | | ⊕⊝⊝⊝ VERY LOW ^1^ | | It was not possible to pool these data due to the way they were reported.  The evidence on the effect of sfSRS on health-related quality of life is very uncertain. |
| ***The risk in the intervention group** (and its 95% CI) is based on the assumed risk in the comparison group and the **relative effect** of the intervention (and its 95% CI).  a: only grade 1 corresponds to normal facial function, the remaining grades 2 to 6 were operationalized as facial palsy b: range of 1 (normal hearing) to 10 (completely deaf)  c: considered functionally preserved if grade A or B was maintained d: total score is based on the seven domain scores; each cover a range of values from 0 to 100, and higher values correspond to lower perceived symptom e: total score and the three domain scores each cover a range of values from −100 to 100, and higher values correspond to lower perceived symptom f: range of 0 to 100, higher values correspond to a greater perceived symptom g: two summary scores, MCS and PCS each have a range of values from 0 to 100, higher values correspond to a lower perceived symptom **AAO-HNS:** American Academy of Otolaryngology-Head and Neck Surgery; **CI:** confidence interval; **GBI:** Glasgow Benefit Inventory; **MCS:** Mental Component Summary; **MD**: mean difference; **MR:** microsurgical resection; **PCS:** Physical Component Summary; **OR:** odds ratio; **PANQOL:** Penn Acoustic Neuroma Quality of Life Scale; **SF 36**: 36-Item Short-Form Health Survey; **sfSRS**: single-fraction stereotactic radiosurgery | | | | | | | | | | |
| **GRADE Working Group grades of evidence  High certainty:** We are very confident that the true effect lies close to that of the estimate of the effect.  **Moderate certainty:** We are moderately confident in the effect estimate: the true effect is likely to be close to the estimate of the effect, but there is a possibility that it is substantially different.  **Low certainty:** Our confidence in the effect estimate is limited: the true effect may be substantially different from the estimate of the effect.  **Very low certainty:** We have very little confidence in the effect estimate: the true effect is likely to be substantially different from the estimate of effect. | | | | | | | | | | |
| ^1^Downgraded one level due to risk of bias (failure to adequately control confounding [Myrseth 2009, Pollock 2006] or unclear number or patients for whom a survey was at least partially retrospective [Carlson 2021]). ^2^Graded up one level due to a very large magnitude of effect. | | | | | | | | | | |
